# Getting a balance between generalisation and specialisation in mental health services: a defence of general services

**DOI:** 10.1192/bjb.2018.52

**Published:** 2018-12

**Authors:** Richard Laugharne, Matthew Thompson, Alind Srivastava, Simon Marlow, Rohit Shankar

**Affiliations:** 1 University of Exeter Medical School, UK; 2 Cornwall Partnership NHS Foundation Trust, UK

## Abstract

Mental health services in the UK National Health Service have evolved to include primary-care generalist, secondary-care generalist and secondary-care specialist services. We argue that there continues to be an important role for the secondary-care generalists as they minimise interfaces, can live with diagnostic uncertainty and support continuity of care. The lack of commissioning and clinical boundaries in secondary-care generalist services can undermine their feasibility, leading to difficulties recruiting and retaining staff. There is a risk of a polo-mint service, where the specialist services on the edge are well resourced, but the secondary-care generalist services taking the greatest burden struggle to recruit and retain clinicians. We need to establish equity in resources and expectations between generalist and specialist mental health services.

**Declaration of interest:**

None.

We all like to feel special. The word ‘generalist’ seems to imply the opposite – ‘Jack of all trades, master of none’. Being a specialist suggests one is at the top of the knowledge tree, whereas the generalist might feel that the term implies that there is someone out there with greater expertise.

And yet … the crowning glory of the British National Health Service (NHS) is the general practitioner (GP). All western countries have specialists, but the central role of the GP in British medicine is a distinctive characteristic of the NHS. There is a high degree of trust between the GP and patient,^1^ created by continuity of care, good therapeutic relationships and a holistic view of the whole patient. The role of the GP creates a degree of efficiency, as those referred to the specialists are screened by doctors who know the patient and ration precious health resources according to need. This may be an idealised picture, but there is still a strong coherent relationship between the GP and patient in the UK. Leinster^2^ argued that medical schools need to concentrate on producing doctors who are good generalists who can orchestrate good care by specialists.

Within UK secondary-care mental health services, there are disparities between general services and specialist services. The latter include specialist services defined by diagnosis or treatment: rehabilitation, early intervention in psychosis, eating disorder, forensic and personality disorder teams. Within secondary-care mental health general services, there can be specialised services according to the environment (in-patient wards), crisis teams and community mental health teams.

As a result, it can be argued that a three-tier service structure has developed, in terms of medical treatment of mental illness in the NHS (Table 1). Some areas straddle the secondary-care generalist and specialist categories, such as forensic teams who are accessed through secondary-care generalists but also through the criminal justice system. We will refer to ‘secondary-care generalist psychiatrists’ as generalists for brevity in this article.

**Table 1:** The three-tier service structure for medical treatment of mental illness in the National Health Service

Medical expertise	Characteristics	Example environments	Patients treated
Primary-care generalists	GPs with broad medical knowledge, open access to the public	General practice	Mild to moderate depression and anxiety
Secondary-care generalist psychiatrists	Psychiatrists working across an age group, usual access by referral from a GP	Psychiatric wards, community mental health teams, crisis teams, liaison teams	Severe mental illness
Secondary-care specialist psychiatrists	Psychiatrists working in narrow diagnostic fields, usual access through secondary-care generalist psychiatrists	Early intervention teams, personality disorder teams, eating disorder teams, rehabilitation/recovery teams	As defined by the team diagnoses

GP, general practitioner.

## Strengths and weaknesses of generalists and specialists in secondary-care psychiatry

Specialists concentrate on a specific diagnosis or treatment of mental illness. In doing so, they develop greater expertise in interventions, which may improve clinical outcomes. There is clear coherent communication to the team and regarding what they are trying to achieve. Often, workload is more precisely commissioned and framed by inclusion criteria and case-load limits. Evidence suggests experts may be better at detecting clinical errors in their field.^3^

Generalist psychiatrists take responsibility for patients across a broad group of diagnoses and often across different environments. Their strength is in their versatility, working across situations, with diagnostic uncertainty and with patients who have more than one diagnosis. As such, they may provide more holistic care, and interfaces between services are less likely to hinder care or waste time. Continuity of care and therapeutic relationships may be easier to establish.

The weaknesses of specialisation include interfaces between teams, which are often inefficient. Working with one group of patients can get boring for staff and they can miss the variety that comes with being a generalist. There is evidence that specialists ‘pull’ cases toward their specialism and are less flexible in their thinking. Their specialism can lead to a bias in their clinical reasoning.^4^

The weaknesses of generalists mirror the strengths of the specialist in that, because they are doing a variety of interventions less often, they can have less expertise, and this can compromise outcomes. Paediatric heart surgery is an example of specialist teams having better outcomes than general heart surgeons in the cardiac care of children. Generalists may neglect certain diagnoses that are difficult to treat and provide a service skewed toward their own interests.

## A defence of generalist services

### The issue of diagnostic uncertainty and avoiding difficult patients

A difficulty in mental health is that diagnostic boundaries are not so sharp that competent colleagues may disagree. For example, there can be differences in defining psychosis from non-psychosis, leading to disagreements as to individual suitability for the early intervention teams. The distinction between bipolar type II disorder and emotionally unstable personality disorder is grey, so that many patients meet diagnostic criteria for both. Dual diagnoses are common, for example, between depression and personality disorder, and psychosis and substance misuse. It is human nature that if a particular patient is challenging to treat, we see aspects of their presentation that exclude them from our responsibility and make them suitable for another team.

### The pain of interfaces

The resulting disagreements between multiple teams are distressing for patients, inefficient and cause resentment between staff, in that referrers have to convince the specialist team that a patient is appropriate. The fewer interfaces the better, and if different staff members know each other and have a relationship, these problems are easier. For a successful service, therapeutic relationships between staff are as important as the therapeutic relationships between patients and clinicians.

### The inequitable distortion of resource allocation

Specialist services often have more tightly defined boundaries than general services and are often commissioned with a defined capacity. For example, specialist teams may have a cap on individual clinician case-loads (e.g. 12–15 in early intervention services) whereas generic community mental health teams (CMHTs) often do not, and case-loads can increase to over 40, with no managerial definition of a reasonable case-load despite the same team taking and assessing most referrals from primary care. The lack of a ‘lid’ on capacity in generalist CMHTs can lead to staff burnout, difficulties in retention and unsafe services.

### The risk of the polo-mint service

Commissioners can prioritise specialist teams over generic teams and skew the service. When the more specialist teams have a protected case-load size and patient number, they can effectively become a local tertiary service. Staff can observe that clinicians have the time and resource in the specialist teams to deliver better quality care that they do not have in generic teams on the wards and in the CMHTs, and apply for specialist posts. This can result in a polo-mint service: posts are filled in specialist teams, which do not take on the immediate burden of new assessments, admissions and the ongoing care of the majority of the patients. Generic teams can be staffed with less-experienced staff and temporary staff. This is inequitable and breeds resentment. There is good evidence that staff burnout increases levels of sickness absence and staff turnover.^5^

### The needs for specialist teams change over time

In the 1980s the day hospital specialist was popular as the asylums were closing. There are very few day hospitals now. The HIV psychiatrist emerged in the 1990s, but thankfully was soon not needed because of the rise of better treatments for HIV. Specialists in assertive outreach were appointed in the 1990s and 2000s but are now disappearing. In the past decade, addiction specialist posts have been eroded, but liaison psychiatry is experiencing a renaissance with medically unexplained symptoms a rediscovered clinical challenge and patients with dementia in general hospitals also a priority to manage.

So what? The world changes, patients’ needs change and specialisms come and go. That may be a good thing, but it emphasises the need for psychiatrists and mental health clinicians to protect their core generic skills and adapt them to fluid challenges. Being a specialist and protecting your speciality may stifle innovation and generalists have the overview to spot new needs requiring innovation.

## How do we get the balance right?

We offer some scenarios to consider.

### Abolish secondary-care generalist teams and have specialist-only teams

This would be an answer, and has parallels in acute medicine where the role of the general physician has diminished and cardiologists, renal physicians, gastroenterologists and other subspecialists have emerged. This may have led to better treatments and outcomes. However, the care of elderly patients with multiple morbidities has fragmented, and the experience of going to hospital has become socially quite unpleasant because of this fragmentation. Patients often do not have a named consultant or a named nurse.

Both politicians and leaders in the Royal College of Physicians have called for a greater emphasis on generalism.^6^ The former editor of the *BMJ* has stated that doctors and patients are heading in opposite directions, ‘patients have multiple conditions whilst doctors are specialising not just in organ systems but parts of organs’.^7^ In the USA, the Council on Graduate Medical Education recommended an increase in the percentage of generalists among practicing physicians to increase from 32 to 40% in 2010 – not only is this target predicted to be unmet, but the percentage of generalists is likely to fall to 25%.^8^

The biggest difficulty for psychiatry is that the lack of clear delineation between diagnoses and patients could be passed between disagreeing teams. However, it is an option that needs consideration.

### Minimise the use of local specialist teams and return to generalist mental health teams

This does seem somewhat backward, but may be a less-fashionable description of integrated services. After all, general adult psychiatry is a speciality in its own right, so why can't one team treat all diagnoses? Generic psychiatric skills require a holistic biopsychosocial approach and the incorporation of a full multidisciplinary team. The secondary-care generalist psychiatrist does not become deskilled when they are on call as they are used to diagnostic uncertainty and a broad view of aetiology and treatment.

Many specialist teams were introduced because of charismatic proponents claiming better outcomes but without evidence for effectiveness, and subsequent research failed to demonstrate efficacy. Just as assertive outreach teams have waned in the UK because of a lack of evidence of better outcomes, with patients returning to generalist teams, others may go the same way.^9^ Some staff might take on a specialist interest within a generalist team, although it is hard to meet and learn with specialist peers if they are separated between teams.

### Have realistic and equitable estimates for all teams in terms of clinician case-load and team case-load

There needs to be equity in the commissioning of secondary-care mental health generalist and specialist services. Specialist teams often have tightly defined commissioning criteria whereas the criteria for generic CMHTs are not defined. The case-load size for different teams may not need to be equal, but they do need to be justified. Although generic teams exist, it is likely they will be the default service for complex patients with diagnostic uncertainty, and boundaries are harder to define than for specialist teams.

Generic CMHTs have tried to control their workload by defining inclusion and exclusion criteria and treatment pathways. However, commissioning needs to allow for the uncertainty of the interface between primary and secondary care. Patients and GPs get frustrated when criteria are so inflexibly followed that patients who are presenting in an unusual or atypical manner are refused care, or have to get worse before they are offered help. Some patients who are ill and at risk do not follow the pathway or treatments recommended. Mental health services are unique in being asked to be assertive in caring for reluctant patients and forcing care in certain circumstances.

## Some suggestions

We believe that generic general adult psychiatric services are likely to be required because specialist-only services will not have the flexibility to roll with diagnostic uncertainty and changes in patient needs. In the past 30 years, new treatments have emerged for emotionally unstable personality disorder, which was once a diagnosis of exclusion and adult attention-deficit hyperactivity disorder, which was completely ignored (and still is in some places). Patients with high-functioning autism still tend to be pushed away, but treatments may emerge. Generalists are flexible and can think on their feet.

Somehow, we need to make working in a generalist CMHT attractive again. This may be through better management of expectations and case-load, career opportunities, pay or quality of life. We cannot lose good staff to specialist teams and leave more junior staff doing demanding generalist jobs.

Currently, the most pressing need is for equity between generic and specialist teams in terms of defined case-load. Staff/patient ratios are a cause of burnout, which increases staff turnover.^5^ Staff in generic CMHTs must have boundaries put on their patient case-load in the same way that specialist teams define their capacity. This does not have to be equal – it may be 35 cases compared with 15 for teams with more intensive input. There have been attempts to develop tools to promote equity between teams in Australia.^10^ Government policy and commissioners cannot continue to be inequitable in their expectations of generic and specialist teams, and as long as CMHTs are treated as inexhaustible, they will not retain the skilled clinicians they need.

## About the authors

**Richard Laugharne** (FRCPsych) is a consultant psychiatrist at Cornwall Partnership NHS Foundation Trust, UK, and Honorary Senior Lecturer at University of Exeter Medical School, UK. **Matthew Thompson** (MRCPsych) is an associate specialist psychiatrist at Cornwall Partnership NHS Foundation Trust, UK. **Alind Srivastava** (MRCPsych) and **Simon Marlow** (MRCPsych) are consultant psychiatrists at Cornwall Partnership NHS Foundation Trust, UK. **Rohit Shankar** (FRCPsych MBE) is a consultant psychiatrist at Cornwall Partnership NHS Foundation Trust, UK, and Honorary Senior Lecturer at University of Exeter Medical School, UK.
